# *Trichinella spiralis* Excretory–Secretory Products Induce Tolerogenic Properties in Human Dendritic Cells *via* Toll-Like Receptors 2 and 4

**DOI:** 10.3389/fimmu.2018.00011

**Published:** 2018-01-24

**Authors:** Nataša Ilic, Alisa Gruden-Movsesijan, Jelena Cvetkovic, Sergej Tomic, Dragana Bozidar Vucevic, Carmen Aranzamendi, Miodrag Colic, Elena Pinelli, Ljiljana Sofronic-Milosavljevic

**Affiliations:** ^1^Institute for the Application of Nuclear Energy, University of Belgrade, Belgrade, Serbia; ^2^Medical Faculty of the Military Medical Academy, University of Defence, Belgrade, Serbia; ^3^Groningen Biomolecular Science and Biotechnology Institute (GBB), University of Groningen, Groningen, Netherlands; ^4^Centre for Infectious Disease Control Netherlands, National Institute for Public Health and the Environment (RIVM), Bilthoven, Netherlands

**Keywords:** *Trichinella spiralis*, excretory–secretory products, dendritic cells, tolerance, immunomodulation, toll-like receptors

## Abstract

*Trichinella spiralis*, as well as its muscle larvae excretory–secretory products (ES L1), given either alone or *via* dendritic cells (DCs), induce a tolerogenic immune microenvironment in inbred rodents and successfully ameliorate experimental autoimmune encephalomyelitis. ES L1 directs the immunological balance away from T helper (Th)1, toward Th2 and regulatory responses by modulating DCs phenotype. The ultimate goal of our work is to find out if it is possible to translate knowledge obtained in animal model to humans and to generate human tolerogenic DCs suitable for therapy of autoimmune diseases through stimulation with ES L1. Here, the impact of ES L1 on the activation of human monocyte-derived DCs is explored for the first time. Under the influence of ES L1, DCs acquired tolerogenic (semi-matured) phenotype, characterized by low expression of HLA-DR, CD83, and CD86 as well as moderate expression of CD40, along with the unchanged production of interleukin (IL)-12 and elevated production of IL-10 and transforming growth factor (TGF)-β, compared to controls. The interaction with DCs involved toll-like receptors (TLR) 2 and 4, and this interaction was mainly responsible for the phenotypic and functional properties of ES L1-treated DCs. Importantly, ES L1 potentiated Th2 polarizing capacity of DCs, and impaired their allo-stimulatory and Th1/Th17 polarizing properties. Moreover, ES L1-treated DCs promoted the expansion of IL-10- and TGF-β- producing CD4^+^CD25^hi^Foxp3^hi^ T cells in indolamine 2, 3 dioxygenase (IDO)-1-dependent manner and increased the suppressive potential of the primed T cell population. ES L1-treated DCs retained the tolerogenic properties, even after the challenge with different pro-inflammatory stimuli, including those acting *via* TLR3 and, especially TLR4. These results suggest that the induction of tolerogenic properties of DCs through stimulation with ES L1 could represent an innovative approach for the preparation of tolerogenic DC for treatment of inflammatory and autoimmune disorders.

## Introduction

One of the permanent challenges in immunology is overcoming the rising problem of losing the delicate balance, provided by the innate immunity, reflected in responding to foreign antigens while remaining tolerant to self-antigens. If this balance is altered due to an increased inflammatory response and diminished tolerance, it can result in autoimmune diseases such as type I diabetes, multiple sclerosis, rheumatoid arthritis, and inflammatory bowel disease (1). Currently available treatments for those diseases do not provide cure or a long-term remission. They usually include immunosuppressive drugs or biological agents which slow down the disease progress but can cause serious adverse effects (2). Given that available therapy cannot restore self-tolerance and provides only temporary remission, efforts are being made in order to develop new therapeutic approaches that would enable the restoration of tolerogenic immune response and silencing of autoimmune processes (3, 4). Dendritic cells (DCs), key antigen-presenting cells, possess the capacity for a fine tuning of the immune response and represent a good candidate as an immunotherapeutic tool (5–8), since their plasticity provides the opportunity to reverse the autoimmune process by mediating restoration of self-tolerance (9).

From the beginning of twenty-first century, a number of results obtained in animal model systems provided evidence that DCs could be treated in a way to acquire tolerogenic properties and that such DCs have a potential to mitigate autoimmune diseases like autoimmune diabetes (10, 11), collagen-induced arthritis (12, 13), experimental autoimmune encephalomyelitis (EAE) (14–16), experimental autoimmune myasthenia gravis (17, 18), and experimental autoimmune uveoretinitis (19, 20). It has been demonstrated that the tolerogenic status of DCs depends on the applied stimuli and maturation conditions. The doctrine that, by default, immature DCs are considered tolerogenic while mature DCs are immunogenic and induce effector responses (21) has been modified by the fact that even mature DCs could have tolerogenic properties (1, 22). Nevertheless, tolerogenic phenotype of DCs usually refers to semi-matured cells with low to intermediate expression of MHC II, as well as co-stimulatory molecules CD80, CD86, and CD40, with elevated production of interleukin (IL)-10 but decreased production of IL-12 (23–25). Different agents proved to be potent inducers of tolerogenic DCs phenotype. Among them are vitamin D3 (26, 27), corticosteroids (28), rapamycin (29), IL-10 (30, 31), or other cytokines (32). The observed phenomenon that tolerogenic DCs could modulate the course of autoimmune disease in terms of reducing the clinical signs and the severity of the disease, directed research from animal model system toward human DCs. Some of these investigations, that showed success *in vitro*, have turned into phase I clinical trials over time (5, 33). Nevertheless, a search for agents able to induce stable tolerogenic DCs is an ongoing story.

It is well known that parasite antigens have the potential to modulate the host immune response *via* DCs by inducing T helper (Th)2 and regulatory response while simultaneously inhibiting Th1 and Th17 response (34) and some of the investigated parasitic antigens showed the capacity to induce tolerogenic DCs phenotype (35, 36). Still, the results considering the impact of parasitic products on human DCs, their tolerogenic properties and the potential of these tolerogenic DCs to modulate the immune response, as well as the mechanisms employed in this phenomenon, are scarce. Potential candidates for the induction of tolerogenic DCs are excretory–secretory (ES L1) antigens of *Trichinella spiralis* muscle larvae. ES L1 antigens are a complex mixture of molecules, released by this parasite into the circulation during the chronic phase of the infection, which can activate regulatory network elements as guardians of homeostasis. Through the action of these products, mediated mainly by DCs, the parasite suppresses the host immune response against itself in order to survive, but it also mitigates the unwanted immune responses like those to autoantigens and allergens (37). Several studies, including our own (38), preformed in mouse model system, showed that ES L1 antigens of *T. spiralis* muscle larvae, or its components (39) possess the ability to induce the semi-matured DCs, which are able to induce the expansion of regulatory T cells (Tregs) *in vitro*. Our work using the rat model system also demonstrated that upon treatment with ES L1 antigens, DCs acquire semi-mature status and an increased capacity to induce Th2 and regulatory immune response both *in vitro* and *in vivo* (40). Also, ES L1-treated DCs, if applied prophylactically, showed considerable ability to modulate the outcome of EAE in Dark Agouti rats by activating and maintaining anti-inflammatory and regulatory immune response while alleviating pro-inflammatory response (16). This was reflected in the enhanced production of IL-4, IL-10, and transforming growth factor (TGF)-β, as well as in diminished production of interferon (IFN)-γ and IL-17, both on systemic level and in the target tissue (CNS). Also, the data obtained in this study indicated that the increased proportion of Foxp3^+^ Tregs on systemic level and in CNS was associated with the amelioration of EAE. Moreover, the applied DCs managed to maintain such immunological profile throughout the disease, which indicates that ES L1-induced tolerogenic properties of DCs are functionally stable. Those results suggest that the immunomodulatory properties of ES L1-treated DCs are worth further research and the present study was designed to translate the knowledge obtained in animal model system on humans. However, considerable differences in immune system exists between human and rodents (41), especially in DCs populations (42). Therefore, it is critical to investigate whether ES L1 antigens could induce similar tolerogenic properties of human DCs as well. Here, we found for the first time that *T. spiralis* ES L1 antigens indeed possess the ability to establish stable tolerogenic human DCs *in vitro*, which could be potentially useful to modulate autoimmune diseases in humans.

## Materials and Methods

The minimum information about tolerogenic antigen-presenting cells checklist was followed for the preparation of this manuscript (43).

### Ethics Statement

Animal experiments were performed according to institutional guidelines and were approved by the local Institutional Animal Care and Use Committee of the Institute for the Application of Nuclear Energy.

Samples of human peripheral blood were obtained from healthy volunteers after written informed consent in accordance with the Declaration of Helsinki and approval by the Ethical Board of the Institute for the Application of Nuclear Energy.

### Antigen Preparation

Adult male Wistar rats, aged 10–12 weeks, were obtained from Military Medical Academy (Belgrade, Serbia) and were housed under standard conditions in animal room with access to food and water *ad libitum*. The rats were used for maintaining of *T. spiralis* strain (ISS 161). Muscle larvae were recovered by digestion of the carcasses in pre-warmed gastric juice (44), and kept under controlled conditions (37°C, 5% CO_2_) in complete Dulbecco’s modified Eagle medium (DMEM) (Sigma), for 18 h (45). ES L1 antigens were obtained by dialysis and concentration of the culture supernatants to at least 4.2 mg/ml in sterile phosphate buffered saline (PBS). Potential endotoxin contamination in ES L1 antigens was neutralized using SERVA Blue PrepProtein Endotoxin ExMicroKit (AMS Biotechnology, UK) according to the manufactures guidelines. Endotoxin levels in ES L1 preparation, in the highest concentration used in the experiments (200 µg/ml), were lower than 0.5 EU/ml [the limit provided by the US Food and Drug Administration guidelines (46)], as detected by the Limulus Amoebocyte Lysate turbidimetric test. The quality of ES L1 products was checked by *Trichinella* ELISA test (INEP, Serbia). Namely, ES L1 antigens were adhered to microtiter plates and their immunoreactivity was analyzed using reference sera with pre-defined titer of anti-*T. spiralis* specific antibodies, as described previously (47).

### Cell Isolation and Culture

Peripheral blood mononuclear cells (PBMCs) from healthy blood donors were isolated by density gradient centrifugation on Lymphoprep gradient (Carl Roth). The monocytes were isolated by negative magnetic-activated cell sorting (MACS) from PBMCs, using the Monocyte Isolation kit (Myltenil Biotec), following their cultivation in CellGro DC medium (Cell Genix), supplemented with 100 ng/ml of human recombinant GM-CSF and 20 ng/ml of human recombinant IL-4 (both from R&D Systems) (complete DC growth medium). CD3^+^ T cells were isolated from PBMCs by negative MACS, using the Pan T cell isolation kit (Miltenyi Biotec), and they were used as responders in allogeneic coculture experiments with monocyte-derived DCs. The purity of CD14^+^ monocytes and CD3^+^ T cell populations was usually higher than 90%, as evaluated by flow cytometry analysis (CyFlow Cube 6, Sysmex Partec GmbH, Görlitz, Germany) (data not shown).

To differentiate immature DCs, monocytes were cultivated in 24-well plate, 0.5 × 10^6^/well, in the complete DC growth medium for 5 days, refreshing the medium on Day 4. To assess the effect of ES L1 on DCs differentiation, the cells were cultivated during this period in the presence of 10, 50, or 200 µg/ml of ES L1 antigen, or without ES L1 (control DCs). The impact of ES L1 on the maturation of DCs was determined by adding ES L1 antigens to the culture of immature DCs on Day 4, for 48 h. To induce mature DCs, the cells were stimulated with LPS from *Escherichia coli* (500 ng/ml, Sigma-Aldrich) and human recombinant IFN-γ (50 ng/ml, R&D Systems) on Day 5, for the next 24 h. In some experiments, LPS (500 ng/ml) or polyinosininc:polycytidylic acid [Poly (I:C), 10 µg/ml] were used instead of LPS/IFN-γ, to investigate the stability of phenotypical and functional characteristics of DCs acquired after the pulsing with ES L1. To determine the role of toll-like receptors (TLR)2 and TLR4 on the DCs status induced by ES L1, immature DCs were treated with blocking antibodies against TLR2 and/or TLR4 (10 µg/ml each, both from BioLegend), or isotype control antibody (anti-rat IgG, eBioscience), 1 h before the treatment with ES L1. The phenotype of DCs was checked using flow cytometry, whereas DC culture supernatants were used for the cytokines analyses. DCs used for functional assays were extensively washed to prevent the transfer of free ES L1 or stimuli to the cocultures.

### Flow Cytometry

Dendritic cells cultivated with different agents were stained after the culture with the following antibodies/reagents: immunoglobulin (Ig)G1a negative control—peridinin–chlorophyll–protein complex (PerCP), IgG1 negative control—phycoerythrin (PE), IgG1 negative control—fluorescein isothiocyanate (FITC), IgG1a negative control—PE cyanine (Cy)5, IgG1 negative control—allophycocyanin (APC), anti-CD83-FITC, anti-CD86-PE, anti CD40-APC, anti-CCR7-FITC, anti-TGF-β-PeCy5, anti-TLR4:PeCy5 (eBioscience), anti-CD1a-PE (Biolegend), anti-IL12p40/p70-PE, anti-indolamine 2,3 dioxygenase (IDO)-1-APC, anti-Ig-like transcript (ILT)3-PE (R&D Systems), anti-IL-10-FITC (AbD Serotec), anti-CD14-FITC, and anti-HLA-DR-PerCP (Miltenyi). The viability of DCs after the culture with ES L1 antigens was determined by 7-aminoactinomycin D (7AAD; Invitrogen) staining. For T cells staining, the following antibodies were used: anti-forkhead box (Fox)P3-PE, anti-CD4-FITC, anti-CD4-PeCy5, anti-TGF-β-PE, anti-IL-4-PE, anti-IL-10-PE (eBioscience), anti-CD25-PeCy5 (BD Pharmigen), anti-IL-10-FITC (AbD Serotec), IL-17A-PerCP (Biolegend), anti-IFNγ-FITC (R&D Systems), and appropriate negative isotype controls, as indicated.

For surface labeling, the cells were washed once in PBS containing 2% FCS and 0.1% Na-azide and then incubated with primary Abs for 30–60 min at 4°C. Intracellular staining was conducted after the surface staining, by using the flow cytometry fixation and permeabilization kit I (R&D). Intracellular staining of IFN-γ, IL-4, IL-10, IL-17, and TGF-β in T cells, was carried out after the 4-h activation of T cells with phorbol-12-myristate-13-acetate (20 ng/ml, PMA) and ionomycin (500 ng/ml) in the presence of monensin (2 µM). For each analysis, more than 5,000 cells were gated according to their specific side-scatter (SSC)/forward-scatter (FSC) properties, thereby avoiding the cells with low SSC/FSC properties (predominantly dead cells), as indicated. Signal overlap between the FL channels was compensated before each experiment using single labeled cells, and non-specific fluorescence was determined by using the appropriate isotype control antibodies and fluorescence minus one controls.

### T Cell Proliferation Assay

In mixed leukocyte reaction assays, DCs treated with the different stimuli, as well as non-treated DCs, were cultured with MACS-purified allogenic T cells in complete RPMI 1640 medium containing 10% FCS (Capricorn Scientific), l-glutamine, 2-mercaptoethanol (50 µM, Sigma), and antibiotics (penicillin, streptomycin, gentamicin, 1% each, ICN Galenika). T cells (1 × 10^5^/well of 96-well plate) were cocultivated with different number of DCs (0.5 × 10^4^, 0.25 × 10^4^, 0.125 × 10^4^) for 5 days in 96-well round-bottom plates. The blank controls were T cells cultivated separately. To measure the level of T cell proliferation, the cells were pulsed for the last 18 h of coculture with 3H-thymidine (1 μCi/well, Amersham). The radioactivity was measured by β-scintillation counting (LKB-1219 Rackbeta, Finland).

### Activation of T Cells by Allogenic DCs

The capacity of ES L1-stimulated DCs to instruct the polarization of T cells was analyzed in allogenic stimulation assay. DCs (0.5 × 10^4^/well, 96-well round-bottom plate) were cultivated with naive allogenic T lymphocytes in 1:20 ratio, for 6 days. To detect cytokines in the supernatants of allogenic stimulation assay, DC/T cell cocultures were treated with PMA (20 ng/ml) and Ionomicin (500 ng/ml) for the last 4 h of coculture, followed by the harvesting of cell-free supernatants. The levels of cytokines produced in DCs cultures (IL-10, IL-12p70, TGF-β), as well as in DC/T cell cocultures (IL-4, IL-10, IFN-γ, IL-17, TGF-β), were measured in cell-free supernatants by sandwich ELISA Kits (R&D). Additionally, T cells were primed with DCs (2 × 10^3^/well of 96-well round-bottom plate) at a 1:50 DC/T cell ratio for 3 days and then expanded with IL-2 (2 ng/ml, R&D) for two more days (T primed cells, Tpr). In some experiments 1-methylthryptophane (1-MT, 0.5 mM, Sigma), as an IDO-1 inhibitor (48), was added in the priming cocultures to assess the role of IDO-1 in the induction of Tregs. The Tregs were identified by flow cytometry based on their expression of CD4, CD25, FoxP3, IL-10, and TGF-β by flow cytometry.

### Suppressor Assay

T cells primed with DCs (Tpr.) were extensively washed and then cocultivated with allogeneic PBMCs responder cells. PBMCs, 1 × 10^5^ cells/well, were cocultured in 1:2 and 1:4 cell ratios with Tpr. (0.5 × 10^5^ and 0.25 × 10^5^ cells/well, respectively), in 96-well round-bottom plates. In order to assess the proliferation of responder cells, PBMCs were pre-stained with 1 µM CFSE (Invitrogen) for 20 min, at 37°C, at density of 1 × 10^7^ cells/ml. The proliferation of cells was stimulated with 8 µg/ml phytohemaglutinine (PHA), and after 5 days of coculture, the cells were harvested, stained with 20 µg/ml propidium iodide to exclude dead cells from the analysis, and the proliferation of responder T cells was measured by flow cytometry. The Proliferation Index (PI), i.e., the average number of cells derived from the initial cell, was calculated using proliferation fit statistics in FCS Express 4 (*De Novo* Software, Glendale, CA, USA).

### Investigation of Pattern Recognition Receptors (PRRs) Activation by ES L1

HEK-Blue™ reporter cell lines expressing either TLR2, TLR3, TLR4, TLR5, TLR7, nucleotide-binding oligomerization domain-like receptors (NOD)1, or NOD2 (InvivoGen, San Diego, CA, USA) (49) were used to evaluate the potential activation of these PRRs by ES L1. The stimulation of HEK-Blue™ cells by PRR agonists triggers the intracellular signaling events leading to the activation of nuclear factor (NF)-κB and activating protein (AP)-1 and the production of secreted alkaline phosphatase (SEAP). Therefore, the SEAP activities in supernatant correspond to the activation of the specific PRRs.

HEK-Blue™ hTLR2 (hkb-htlr2), HEK-Blue™ hTLR3 (hkb-htlr3), HEK-Blue™ hTLR4 (hkb-htlr4), HEK-Blue™ hTLR5 (hkb-htlr5), HEK-Blue™ hTLR7 (hkb-htlr7), HEK-Blue™ hNOD1 (hkb-hnod1), and HEK-Blue™ hNOD2 (hkb-hnod2) cells were cultured in Dulbecco’s Modified Eagle’s culture medium (DMEM; Gibco, Thermo Fisher Scientific), supplemented with 100 U/ml penicillin, 100 µg/ml streptomycin, 0.3 mg/ml l-glutamine (Gibco; Pen–Strep–Glu 100×; Thermo Fisher Scientific, Rockford, IL, USA) and 10% (v/v) heat-inactivated HyClone FBS (30 min at 56°C) (Thermo Fisher Scientific) at 37°C in 5% CO_2_/95% air. Cell lines (2.5 × 10^4^/well, 96-well flat-bottom plates) were cultivated in 150 µl of culture medium. Cells were treated with 50 µl of various concentrations of ES L1 antigens (50, 5, and 0.05 µg/ml) or PRRs agonists: Pam3CSK4 (10 ng/ml) as TLR2 ligand, Poly (I:C) HMW (1 µg/ml) as TLR3 ligand, LPS (1 ng/ml) *E. coli K12* as TLR4 ligand, FLA-ST (50 ng/ml) as TLR5 ligand, Imiquimod (10 µg/ml) as TLR7 ligand, iE-DAP (25 µg/ml) as NOD1 ligand, and MDP (10 µg/ml) as NOD2 ligand (all from InvivoGen). After 22–24 h incubation, supernatants were collected for determination of SEAP activity. QUANTI-Blue™ medium (180 µl) substrate and cell culture supernatant (20 µl) were added per well on a flat-bottom 96-well ELISA plate. After 4 and 24 h of incubation at 37°C in 5% CO_2_, the levels of SEAP activity were analyzed by measuring the absorbance at 649 nm on a BioTek EL808 microplate reader and Gen5 Data Analysis Software (BioTek, VT, USA).

### Statistical Analyses

Data are presented either from representative experiments or as mean ± SD of at least three experiments carried out with different DCs donors. Statistical analysis of the data was conducted using repeated measures ANOVA, followed by a Tukey’s posttest in PRISM5 (Graphpad software), and for *p* < 0.05, the differences were considered significant statistically.

## Results

### ES L1 Antigens Impair the Maturation of DCs, without Affecting Their Differentiation

**Figure 1:**
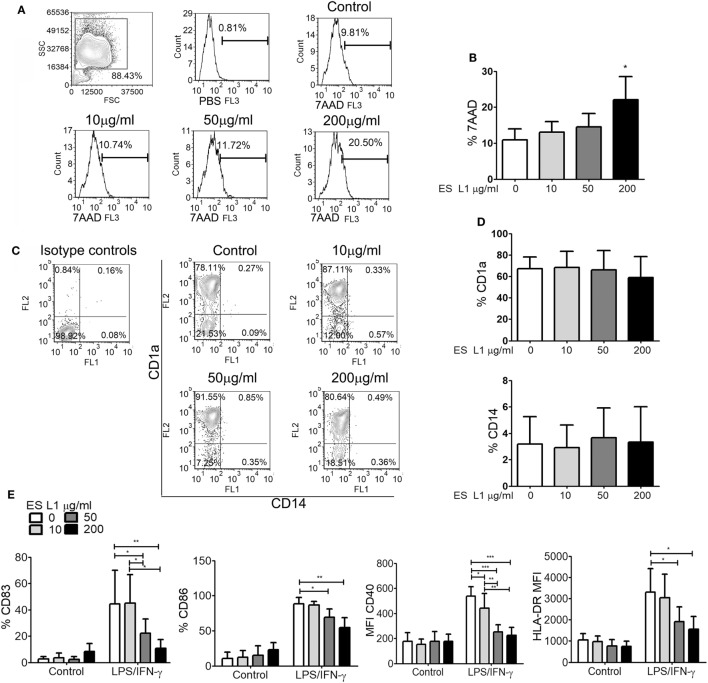
Effects ES L1 antigens on differentiation of dendritic cells (DCs). **(A–E)** Immature DCs were generated from monocytes in GM-CSF/interleukin-4 supplemented medium in the presence of ES L1 (10, 50, and 200 µg/ml) antigens, or their absence (control), during 5 days. **(A)** Representative histograms from the analysis of 7AAD^+^ (dead) cells after the culture are shown and, **(B)** the summarized results from three different donors are shown as mean% ± SD. **(C)** Representative plots for CD1a and CD14 expression on DCs after 5 days of culture with ES L1 are shown, and **(D)** the summarized results from three different donors are presented as mean% ± SD. **(E)** DCs differentiated in the presence or absence of ES L1, were stimulated with LPS/IFN-γ on Day 5, and the expression of CD83, CD86, CD40, and HLA-DR was analyzed by flow cytometry after 24 h. The results collected with three different DC donors are shown as mean ± SD (see also Figure S1 in Supplementary Material, showing a representative experiment). *p < 0.05, **p < 0.01, ***p < 0.005 compared to corresponding control DCs, or as indicated (one-way ANOVA with Tukey’s posttest).

Human monocytes cultured in complete DC growth medium *in vitro* differentiate into immature DCs, by upregulating CD1a and diminishing CD14 expression (50). To assess the influence of ES L1 antigens on differentiation of DCs, monocytes were differentiated with GM-CSF/IL-4 in the presence or absence of different doses of ES L1 antigens (10, 50, or 200 µg/ml), applied on Day 0. To exclude the possibility that the immunomodulatory effects of ES L1 antigens were due to their cytotoxicity, we first analyzed the percentage of dead (7AAD^+^) cells after 4 days of cultivation with ES L1. It was observed that the percentage of 7AAD^+^ DCs cultivated with 10 or 50 µg/ml was similar to the values obtained in control DCs cultures. In contrast, 200 µg/ml ES L1 increased the percentage of 7AAD^+^ cells up to 27% (Figures 1A,B). However, none of the applied ES L1 doses induced more than 30% of dead DCs which, according to ISO 10933-5 (51), could be interpreted as the lack of cytotoxicity. The phenotypic analysis of DCs suggested that ES L1 did not interfere with DCs differentiation irrespective of the dosage applied, since the percentage of CD1a and CD14 on ES L1-treated DCs was similar to that on un-treated control DCs (Figures 1C,D).

To evaluate whether the differentiation of DCs with different ES L1 doses affect their subsequent maturation, DCs were stimulated with LPS/IFN-γ on Day 5 of culture, for the next 24 h. It was observed that differentiation with higher doses of ES L1 (50 or 200 µg/ml) impaired severely the LPS/IFN-γ-induced upregulation of CD83, CD86, CD40, and HLA-DR, whereas the cultivation with 10 µg/ml ES L1 impaired only CD40 upregulation (Figure 1E; Figure S1 in Supplementary Material). Although 200 μg/ml of ES L1 had the strongest effect, it was not significantly different from the effect of 50 µg/ml ES L1.

### Human DCs Acquire Stable Semi-Mature Phenotype upon Stimulation with ES L1 Antigens

**Figure 2:**
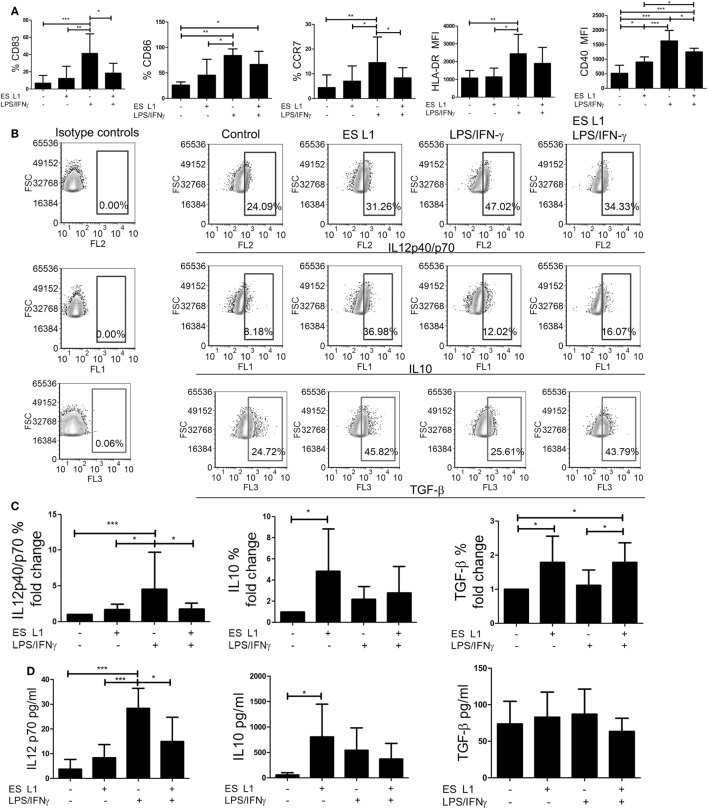
The maturation capacity of dendritic cells (DCs) treated with ES L1 antigens. **(A–D)** Immature DCs were treated with ES L1 antigens (50 µg/ml) on Day 4 of culture for 24 h and then additionally activated or not with LPS/interferon (IFN)-γ for the next 24 h, followed by flow cytometry analysis. **(A)** The percentages of CD83, CD86, HLA-DR, CCR7, and CD40 expression by DCs are shown as mean ± SD from four different experiments (see also Figure S2 in Supplementary Material for a representative experiment). **(B)** Representative analysis of interleukin (IL)-10, IL12p40/p70, and transforming growth factor (TGF)-β expression within DCs are shown, and **(C)** the summarized results are presented as fold change of cytokines expression as mean ± SD from seven different experiments. **(D)** The levels of IL-10, IL-12p70, and TGF-β (picograms/milliliter) in DCs culture supernatants were measured by ELISA test. *p < 0.05, **p < 0.01, ***p < 0.005 compared to control, or as indicated (one-way ANOVA with Tukey’s posttest).

To assess whether ES L1 antigens exhibit similar effects on DCs maturation when added at latter periods of cultivation, the cells were pulsed with ES L1 antigens on Day 4 of cultures and then stimulated with LPS/IFN-γ on Day 5 for the next 24 h, or left un-stimulated. Considering the negligible effects of 10 µg/ml of ES L1 on LPS/IFN-γ-induced DCs maturation and significant immunomodulatory effect of 50 µg/ml of ES L1 without an increased cytotoxicity, further experiments were carried out with 50 µg/ml of ES L1 to monitor the functional significance of ES L1 on DCs maturation. The analysis of maturation markers on DCs, 48 h after the treatment with ES L1, showed that the expression of HLA-DR, CCR7, CD83, and CD86 were somewhat upregulated, but not significantly, compared to the control immature DCs (Figure 2A; Figure S2 in Supplementary Material). ES L1 treatment significantly upregulated only the expression of CD40 (Figure 2A; Figure S2 in Supplementary Material), all of which suggested that ES L1 induce a semi-mature phenotype of DCs. Similar results were obtained when the treatment with ES L1 lasted only 24 h (data not shown). In contrast to ES L1-treated DCs, the expression of all surface markers after the 24h-stimulation with LPS/IFN-γ was significantly upregulated, as expected for type 1 inflammatory DCs induced by this cocktail (52). However, DCs treated with 50 µg/ml of ES L1 before LPS/IFN-γ stimulation displayed an impaired upregulation of maturation markers (Figure 2A), confirming that the effect of ES L1 was limited to the maturation stage of DCs.

To investigate the effect of ES L1 antigens on DCs’ cytokines production, the percentages of IL-10, IL-12, and TGF-β positive DCs were determined by intracellular staining (Figures 2B,C), and the cytokine levels were analyzed in DCs culture supernatants (Figure 2D). Both analyses showed that the levels of IL-12 in ES L1-treated DCs were unchanged compared to control immature DCs, i.e., ES L1 failed to induce the production of IL-12 by DCs. On the other hand, ES L1 significantly increased the percentage of IL-10^+^ DCs and the levels of IL-10 in DCs culture supernatants (Figures 2C,D). The percentage of TGF-β^+^ DCs also increased significantly after the ES L1 treatment, compared to control DCs, but this was not followed by increased levels of TGF-β in the culture supernatants (Figures 2B–D).

To investigate the functional stability of ES L1-treated DCs, the cells were challenged with different maturation stimuli 24 h after the treatment with ES L1 antigens. The stimulation of DCs with LPS/IFN-γ for 24 h, expectedly, induced a strong upregulation of surface maturation markers (Figure 2A; Figure S2 in Supplementary Material), IL-12 expression, and its production by DCs. However no such upregulation occurred if DCs were treated with ES L1 before the challenge with LPS/IFN-γ (Figures 2B–D). The expression of TGF-β in ES L1-treated DCs, after the challenge with LPS/IFN-γ, remained significantly higher compared to control LPS/IFN-γ-matured DCs. Curiously enough, the levels of TGF-β in DCs culture supernatants did not correspond to the percentages of DCs expressing TGF-β intracellularly, which could be a consequence of the autocrine uptake of the produced TGF-β (53). On the other hand, the expression of IL-10 by ES L1-treated DCs matured with LPS/IFN-γ and its production levels were not significantly different from the values obtained with control LPS/IFN-γ-matured DCs (Figures 2B–D).

Considering the inhibitory effects of IFN-γ on IL-10 production by DCs (54), we wondered whether the use of LPS alone, as a maturation stimulus, would exhibit different effects on the phenotype and cytokines production by ES L1-treated DCs. ES L1-treated DCs exhibited similar resistance to LPS-induced phenotypic maturation and IL-12 expression, as when LPS/IFN-γ was used as a stimulus, while the expression of IL-10 in ES L1-treated LPS-matured DCs was significantly higher compared to control LPS-matured DCs (Figure S3 in Supplementary Material). The mitigated effect of LPS/IFN-γ or LPS alone on the phenotype and cytokine production by ES L1-treated DCs was not a consequence of ES L1-induced downregulation of TLR4 on DCs, since its expression was not changed significantly 48 h after the ES L1 treatment (Figure S4 in Supplementary Material).

Since LPS and LPS/IFN-γ stimulation includes a TLR4-dependent induction of DCs maturation (55), we also tested whether ES L1-treated DCs display similar maturation resistance to other TLR stimuli, such as the stimulation *via* TLR3 with its agonist, Poly (I:C). The data showed that ES L1 impaired significantly the upregulation of HLA-DR, CD40, and IL-12 by DCs, but not CD83 and CD86, upon the Poly (I:C) stimulation. Additionally, ES L1-treated Poly (I:C)-matured DCs produced significantly higher levels of IL-10 compared to control Poly (I:C)-matured DCs (Figure S5 in Supplementary Material). These data suggested that the treatment of DCs with ES L1 impeded full maturation of DCs by different maturation stimuli, showing the stability of ES L1-treated DCs.

### DCs Treated with ES L1 Acquire Limited Allo-Stimulatory Capacity and Provoke Th2 and Regulatory Responses

**Figure 3:**
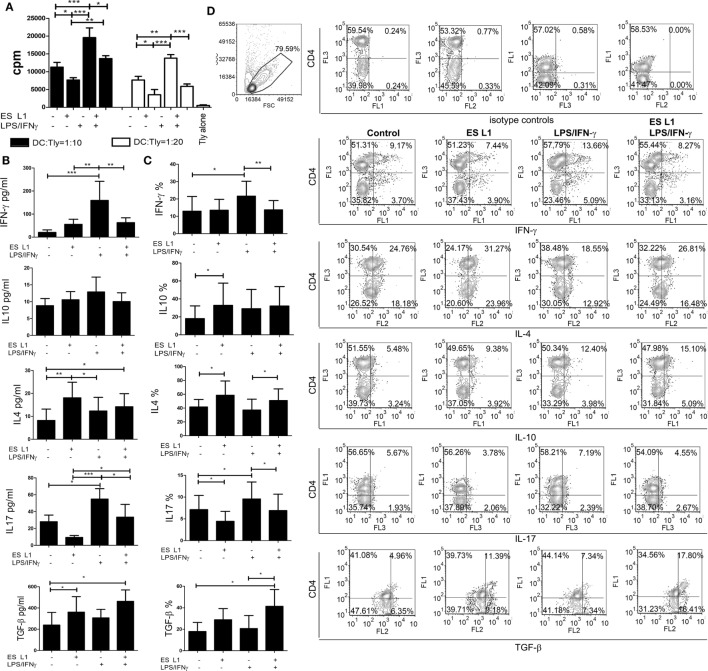
Allo-stimulatory and T helper polarizing capacity of ES L1-pulsed dendritic cells (DCs). **(A–D)** DCs treated with ES L1 antigens (50 µg/ml) and/or LPS/interferon (IFN)-γ were washed thoroughly and then cocultured with magnetic-activated cell sorting-purified allogenic T cells (Tly) (1 × 10^5^/well) for 6 days in two DC:T cell ratios (1:10 and 1:20). **(A)** The proliferation in cocultures was measured by 3H-thymidin incorporation assay, and Tly cultivated alone were used as a blank control. **(B)** The concentration of indicated cytokines were determined in the supernatants of PMA/ionophore-treated DC:T cell cocultures at 1:20 cell-to-cell ratio, respectively, by specific ELISA tests. **(C)** The percentage of cytokines expression measured intracellularly by flow cytometry, within the T cells cocultivated with DCs as in **(B)** and treated with PMA/Ionophore/monensin for the last 4 h, are shown as mean% ± SD of four experiments with different DCs donors. *p < 0.05, **p < 0.01, ***p < 0.001 compared to control, or as indicated by line (one-way ANOVA with Tukey’s posttest). **(D)** The analysis of the intracellular cytokines in T cells was carried out after the surface staining of CD4, as indicated on representative dot plots collected from two experiments.

To assess the capacity of human DCs to influence the proliferation of T cells after the treatment with ES L1, coculture of DCs and allogeneic MACS-purified CD3^+^ T cells was performed, and the T cell proliferation was determined based on 3H-thymidine incorporation level. ES L1-treated DCs had a weaker capacity to stimulate T cell proliferation compared to both immature and LPS/IFN-γ-matured DCs (Figure 3A). The limited T cell proliferation capacity was preserved even when ES L1-treated DCs were challenged with LPS/IFN-γ.

The Th polarization capacity of ES L1-treated DCs was assessed by measuring the levels of IFN-γ, IL-17, IL-4, IL-10, and TGF-β in DCs/T cell cocultures (Figure 3B) and by analyzing the expression of these cytokines within gated CD4^+^ and CD4^−^ (more than 98% CD8^+^) T cell population (Figures 3C,D) after the stimulation with PMA/ionophore. The results suggested that ES L1 potentiated DC-mediated induction of Th2 and regulatory type of immune response, as judged by the increased IL-4, IL-10, and TGF-β expression by T cells and their production in DC/T cell cocultures. The increased capacity of ES L1-treated DCs for the induction of regulatory cytokines in T cells was detected in both CD4^+^ and CD8^+^ T cell population (Figure 3D). After the challenge with LPS/IFN-γ, the capacity of ES L1-treated DCs to induce TGF-β-producing T cells increased even more. Their capacity to induce Th2 cells remained significantly higher than the capacity of control LPS/IFN-γ-matured DCs, whereas the capacity to induce IL-10-producing T cells was similar to the capacity of control mature DCs. Additionally, ES L1-treated DCs displayed an impaired potential to induce Th17 cells in the absence of stimuli, and an impaired Th1/Th17 polarization capacity after their challenge with a strong Th1/Th17 polarizing cocktail, LPS/IFN-γ, which was confirmed by the analyses of both soluble products and intracellular cytokines expression in T cells (Figures 3B–D). Taken together, ES L1-treated DCs displayed a clear shift in polarizing capacity, away from Th1/Th17, toward increased regulatory and Th2 type of immune response.

### DCs Pulsed with ES L1 Antigens Have Tolerogenic Properties

**Figure 4:**
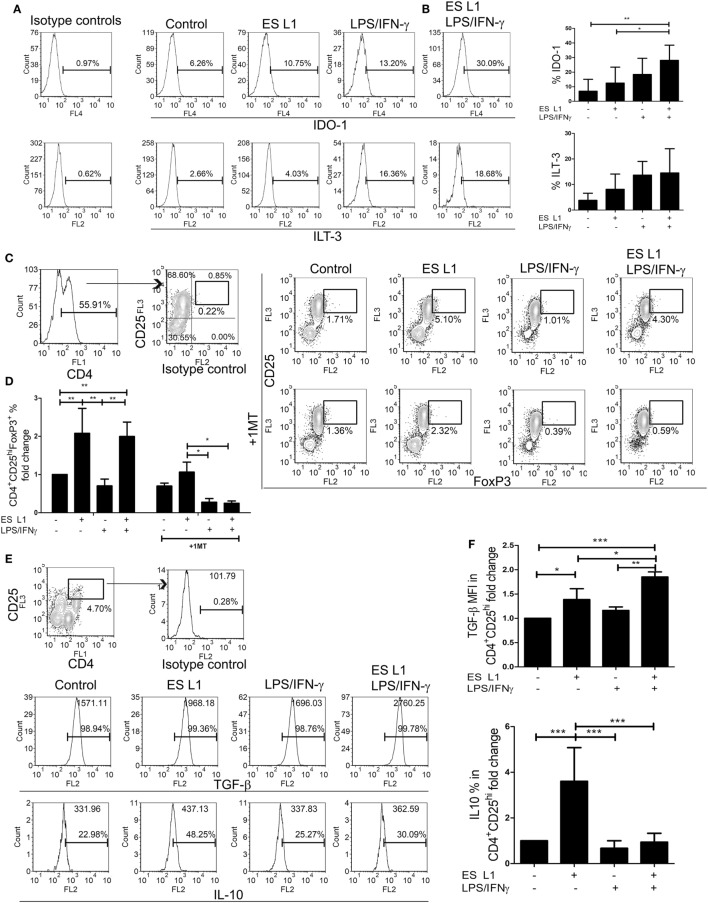
Tolerogenic properties and functions of ES L1-treated dendritic cells (DCs). **(A)** DCs treated with ES L1 antigens (50 µg/ml) and/or LPS/interferon (IFN)-γ, as described, were analyzed for IDO-1 and immunoglobulin-like transcript (ILT)-3 expression, as indicated on representative histograms. **(B)** The summarized results on IDO-1 and ILT-3 expression on three different DC donors is shown as mean% ± SD. **(C)** DCs treated as in **(A)** were washed thoroughly and then cocultivated with magnetic-activated cell sorting-purified allogenic T cells (1 × 10^5^/well) (DC:T cell ratio 1:50), for 3 days and then re-stimulated with interleukin (IL)-2 (2 ng/ml) for another 3 days, all in the presence or absence of 1-MT (0.5 mM). Representative analysis of CD25^hi^FoxP3^hi^ cells within CD4^+^ T cells from one experiment is shown, and **(D)** the summarized results are shown as the mean fold change in regulatory T cells % ± SD from four different experiments. **(E)** Representative analysis of transforming growth factor (TGF)-β and IL-10 within CD4^+^CD25^hi^ T cell population is shown, and **(F)** the summarized results collected from four different experiments are shown as mean fold change in mean fluorescence intensity (MFI) (TGF-β) or % (IL-10) within CD4^+^CD25^hi^ T cell population ± SD. *p < 0.05, **p < 0.01, ***p < 0.005 compared as indicated by line (one-way ANOVA with Tukey’s posttest).

Since DCs upon the treatment with ES L1 antigens acquire semi-mature phenotype, limited allo-stimulatory capacity, and shift the immune response toward Th2 and regulatory type, we presumed that ES L1-treated DCs possess tolerogenic properties. In addition to reduced pro-inflammatory phenotype and functions, the tolerogenic DCs express tolerogenic markers, such as IDO-1 and ILT-3, and display an increased capacity to induce Tregs (56, 57). Therefore, we first analyzed the expression of IDO-1 and ILT-3 by DCs under the influence of ES L1 antigens, in the presence or absence of additional stimulation with LPS/IFN-γ (Figures 4A,B). The results showed that ES L1 induce the expression of both markers on DCs, but not significantly. However, the expression of IDO-1 was significantly increased after the challenge of ES L1-treated DCs with LPS/IFN-γ. The expression pattern of ILT-3 showed similar trend, but the differences were not significant statistically.

The percentage of Tregs in coculture with DCs was determined based on their high expression of CD25 and FoxP3 within CD4^+^ T cells, to distinguish them from transiently activated CD25^+^Foxp3^+^ Th cells. ES L1-treated DCs induced a significant increase in the percentage of CD4^+^CD25^hi^Foxp3^hi^ compared to control cells (twofold), and retained that ability even after LPS/IFN-γ challenge (Figures 4C,D). The observed expansion of Tregs was IDO-1 dependent, as it was demonstrated that the addition of 1-MT in the cocultures decreased significantly the percentages of CD4^+^CD25^hi^Foxp3^hi^ cells (Figures 4C,D). All CD4^+^CD25^hi^ T cells were positive for TGF-β, but it was found that ES L1-treated DCs significantly increased the expression of TGF-β within CD4^+^CD25^hi^ T cells, compared to the control DCs, and this ability of ES L1-treated DCs was even more pronounced after their challenge with LPS/IFN-γ (Figures 4E,F). Moreover, the expression of IL-10 within CD4^+^CD25^hi^ T cell population was fourfold higher upon priming with ES L1-treated DCs than after the priming with control DCs. However, such an upregulation of IL-10 within CD4^+^CD25^hi^ T cells was not observed when DCs were stimulated additionally with LPS/IFN-γ (Figures 4E,F).

Taken together, the above results suggested that ES L1 indeed potentiated the tolerogenic phenotype and functions of DCs, which induced Tregs in IDO-1-dependent manner, especially after the challenge with LPS/IFN-γ.

### T Cells Primed by ES L1-Treated DCs Exhibit Suppressive Activity

**Figure 5:**
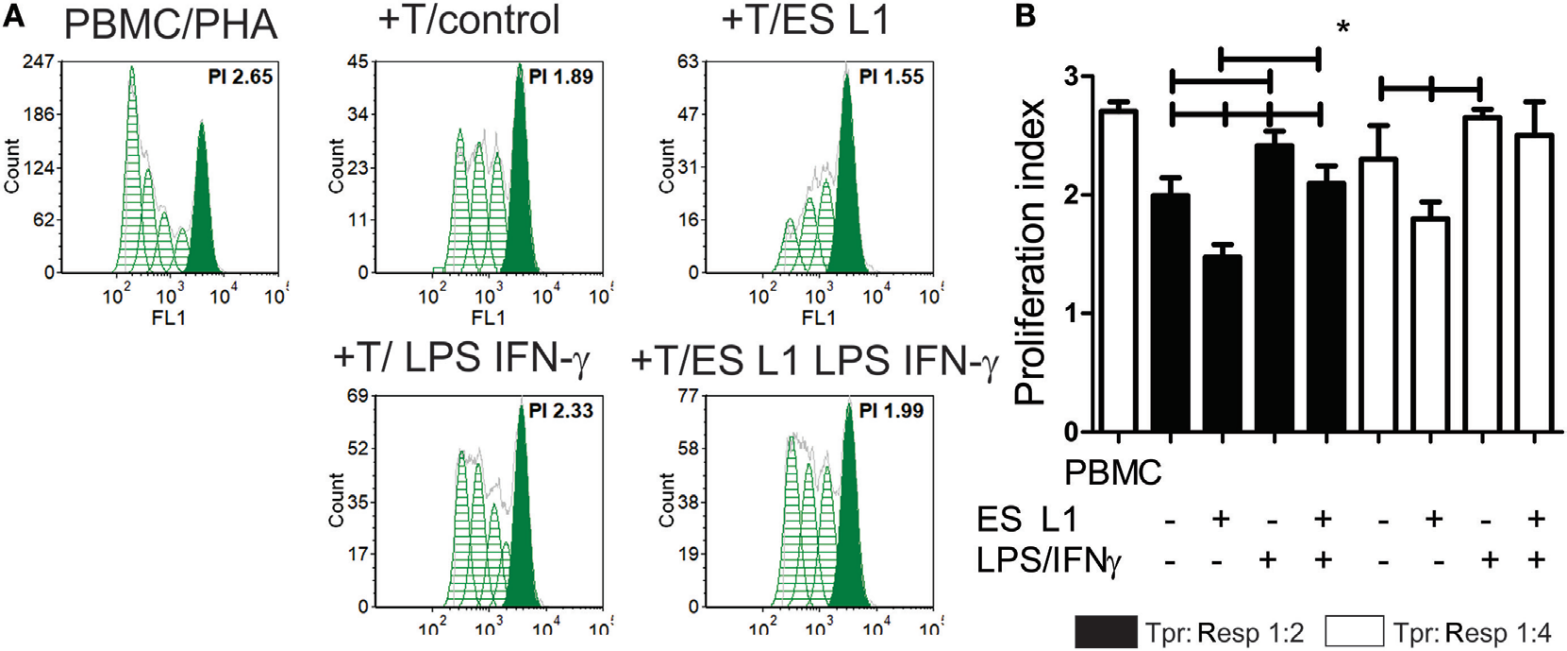
The suppressive potential of T cells primed with ES L1-pulsed dendritic cells (DCs). **(A,B)** magnetic-activated cell sorting-purified CD3^+^ T cells (0.5 × 10^4^ or 0.25 × 10^4^ cells/well) primed with DCs (Tpr.), as described, were harvested after 6 days, washed, and then cocultivated with third-part allogeneic CFSE-labeled Peripheral blood mononuclear cells (PBMCs) (Resp.) (1 × 10^4^ cells) in 1:2 and 1:4 cell-to-cell ratio, respectively, in the presence of phytohemaglutinine (PHA) (8 µg/ml) for the next 5 days. **(A)** Representative histograms from one experiment, with Tpr:Resp. 1:2 cell ratio, is shown, and **(B)** the summarized results from four different experiments are shown as mean PI (the average number of cells derived from the initial cell) of Resp. ± SD. *p < 0.05, compared as indicated by line (one-way ANOVA with Tukey’s posttest).

To investigate whether an increased percentage of CD4^+^CD25^hi^Foxp3^hi^ Treg have any functional significance, the T cell population primed with DCs (Tpr) was cocultivated with CFSE-labeled PBMCs (responders) in the presence of PHA. A flow cytometry analysis of the responder cells’ proliferation clearly suggested that T cells primed with ES L1-treated DCs exhibited a stronger capacity to inhibit the proliferation of responder cells in both 1:2 and 1:4 Tpr:responder cell ratios (Figures 5A,B). Similar results were obtained with T cells primed with LPS/IFN-γ-stimulated ES L1-treated DCs, as demonstrated by their increased potential to suppress the responder cells proliferation in 1:2 cell-to-cell ratio. The results suggested that ES L1-treated DCs retained the ability to induce suppressive T cell populations even in inflammatory environment. Since the percentage of Tregs increased significantly after the priming with ES L1-treated DCs, the observed suppressive activity of the primed T cell population may be ascribed to the effects of Tregs as well.

### ES L1 Antigen Activates TLR2 and TLR4

**Figure 6:**
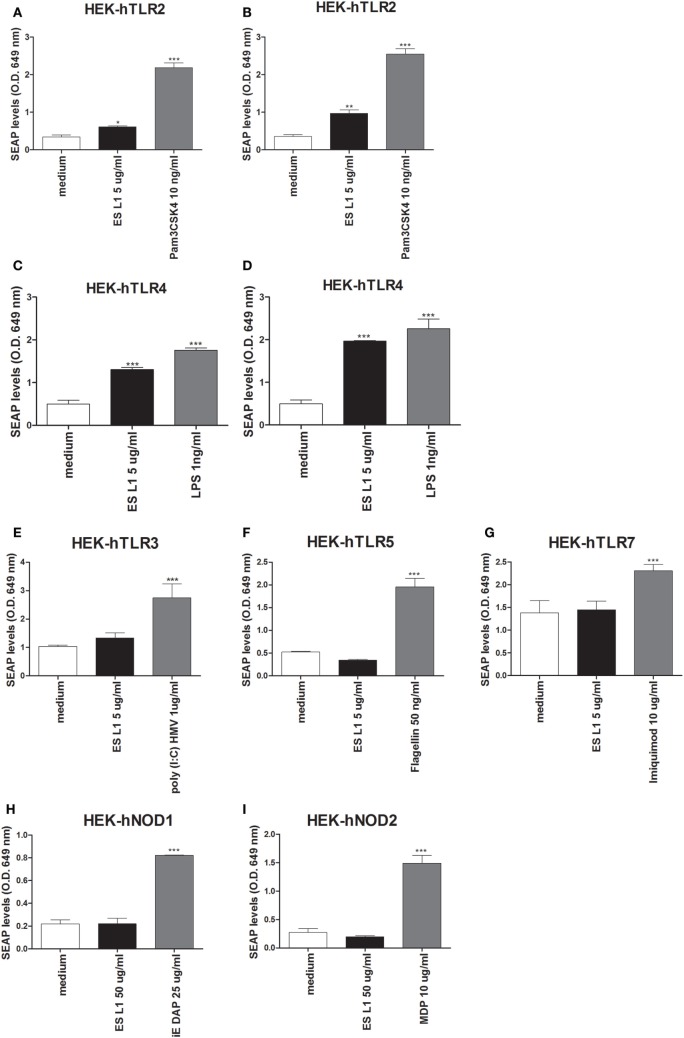
Interaction of ES L1 antigen with pattern recognition receptors (PRRs) on HEK-Blue™ cell lines. HEK-Blue™ cell lines transfected with a single specific human PRR (TLR2, 3, 4, 5, 7, NOD1, and 2) were treated with ES L1 or PRR agonists for 24 h, followed by the analyses of secreted alkaline phosphatase (SEAP) levels (OD 649 nm) released in culture medium at two time points (4 and 24 h after the substrate addition). Pam3CSK4 (10 ng/ml), ES L1 (5 µg/ml)—incubation period 4 **(A)** and 24 h **(B)**; LPS (1 ng/ml) *Escherichia coli K12*, ES L1 (5 µg/ml)—incubation period 4 **(C)** and 24 h **(D)**; Poly (I:C) HMV (1 µg/ml), ES L1 (5 µg/ml)—incubation period 24 h **(E)**; FLA-ST (50 ng/ml), ES L1 (5 µg/ml)—incubation period 24 h **(F)**; imiquimod (10 µg/ml), ES L1 (5 µg/ml)—incubation period 24 h **(G)**; iE-DAP (25 µg/ml) ES L1 (50 µg/ml)—incubation period 24 h **(H)**; MDP (10 µg/ml), ES L1 (50 µg/ml)—incubation period 24 h **(I)**. Results are shown as mean ± SD from three different experiments *p < 0.05, **p < 0.01, ***p < 0.001 compared with control (medium) (one-way ANOVA with Tukey’s posttest).

The data about the receptors that recognize *T. spiralis* antigens are scarce. Having in mind that PRRs are a key element of the innate immune system and have important role in detection of pathogens and subsequent activation of DCs, the interaction between different PRRs (TLRs and NODs) with ES L1 antigens was investigated. The study was performed on HEK-Blue™ reporter cell lines expressing the individual TLRs (TLR2, -3, -4, -5, -7), NOD1, or NOD2 receptors. The activation of PRRs was indicated by SEAP activation in culture supernatants of stimulated cells. All used HEK-Blue™ cell lines were treated with ES L1 antigens, while the cells pulsed with corresponding TLR or NOD agonists were used as positive controls, and the cells cultivated only in medium were used as negative control (Figure 6).

Stimulation of HEK-hTLR2 cells with ES L1 induced an increased level of SEAP activity after 4 and 24 h (Figures 6A,B respectively) compared to negative control, indicating that ES L1 activates TLR2-mediated signaling events. ES L1 treatment of HEK-hTLR4 cells resulted in a significantly increased level of alkaline phosphatase activity after 4 h (Figure 6C) compared to control, and after 24 h (Figure 6D), the level of enzyme activity was similar to the one observed with TLR4 agonist (LPS, 1 ng/ml), suggesting that ES L1 also activated TLR4. HEK-hTLR3, HEK-hTLR5, HEK-hTLR7, HEK-hNOD1, and HEK-hNOD2 cell lines cultivated in the presence of ES L1 antigens, after 4 h (data not shown) and after 24 h, showed no statistically significant change in SEAP activity, compared to control cells, unlike the cells treated with specific PRR ligands as described (Figures 6E–I). The obtained results suggested strongly that ES L1 antigens engage TLR2 and TLR4 and activate NF-κB and AP-1-mediated signaling events by these receptors, while TLR3, TLR5, TLR7, NOD1, and NOD2 receptors do not participate in ES L1-mediated effects.

### TLR2 and TLR4 Are Involved in ES L1-Mediated Induction of Semi-Mature DCs

**Figure 7:**
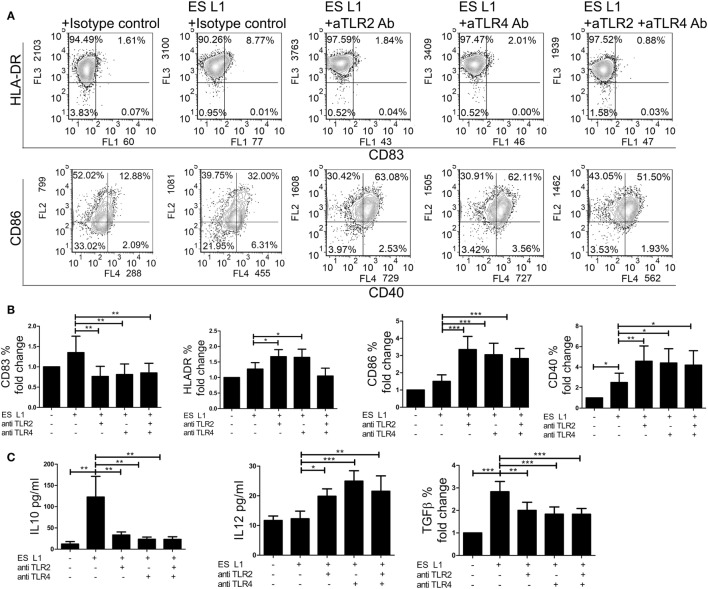
Role of TLR2 and TLR4 in ES L1 antigen-induced dendritic cells (DCs) maturation and cytokine production. **(A–C)** Immature DCs were incubated with ES L1 in the presence of isotype control, or specific TLR2 and/or TLR4 blocking antibodies (10 µg/ml). After 48 h, the expression of surface markers (CD83, CD86, HLA-DR, and CD40) was measured by flow cytometry and the cytokine levels were determined in culture supernatants by ELISA test or by intracellular staining. **(A)** Representative analysis of surface markers expression is shown, and **(B)** the summarized results are shown as fold change in percentage (%) of markers expression ± SD from three different experiments. **(C)** Cytokine levels (picograms/milliliter) for interleukin (IL)-10 and IL-12p70 measured in DCs culture supernatants by ELISA test, and transforming growth factor (TGF)-β expression (% fold change) determined by flow cytometry, are shown as mean ± SD of three different experiments. *p < 0.05, **p < 0.01, ***p < 0.005 as indicated by line (one-way ANOVA with Tukey’s posttest).

Considering that ES L1 antigens activate TLR2 and TLR4, the next step was to investigate the relevance of this interaction for the development of semi-mature DCs by ES L1 antigens. For this purpose, TLR2 and TLR4 receptors were blocked individually or simultaneously using specific monoclonal blocking antibodies, prior to DCs treatment with ES L1 antigens. In the presence of the specific blocking antibodies, the expression of DCs surface markers upon ES L1 treatment was significantly changed compared to control DCs treated with ES L1 antigens in the presence of isotype control antibody. Namely, the expression of CD83 was significantly lower, while CD86, HLA-DR, and CD40 expression were significantly higher compared to control ES L1-treated DCs (Figures 7A,B).

Additionally, the differences in cytokines production by DCs were analyzed in the presence of specific blocking or isotype control antibodies upon the ES L1 treatment (Figure 7C). In case when TLRs were specifically blocked either individually or simultaneously, the anti-inflammatory properties of ES L1-treated DCs were diminished, i.e., the concentration levels of IL-12p70 were significantly higher and the levels of IL-10 and TGF-β were significantly lower compared to those observed in DCs treated with ES L1 and isotype control antibody. The treatment of DCs with isotype control antibody, as well as the blocking of receptors without subsequent treatment with ES L1 antigens, resulted in the same DCs maturation profile as observed with non-stimulated DCs (data not shown). The results obtained by individual blocking of TLR2 and TLR4 indicated that both receptors are involved in ES L1-driven DC phenotype. Simultaneous blocking of two receptors gave no phenotypic or functional changes compared to individual blocking, suggesting the absence of synergistic effect.

## Discussion

A number of studies have dealt with *in vitro* generation of DCs from monocytes that can be manipulated to acquire tolerogenic properties (35, 56, 58–60). These cells were shown to have an immature or semi-mature phenotype, low production of inflammatory and increased production of anti-inflammatory cytokines, the ability to present antigens in a tolerogenic form, and consequently, an increased capacity to induce Tregs. Tolerogenic DCs are considered as a promising tool for development of cell-based therapy applicable in the treatment of autoimmune diseases, chronic inflammation, and transplantation therapy (61). Our previous results obtained on animal model system demonstrated the capacity of *T. spiralis* products to induce the development of DCs with tolerogenic properties (40), which successfully ameliorated autoimmune disease in animal models (16). However, further development of cell therapies based on ES L1 antigens require comprehensive studies on human DCs model system, which have not been conducted so far. Here, we showed for the first time that *T. spiralis* ES L1 antigens represent a promising new tool for the generation of human tolerogenic DCs *in vitro*. In contrast to other protocols for generation of DCs, such as those which include vitamin D3 (26, 27) or IL-10 (31), DCs cultivated with ES L1 antigens from Day 0, even with high doses, displayed an unaltered differentiation pattern, i.e., complete downregulation of CD14 and upregulation of CD1a in the presence of GM-CSF and IL-4. These results suggested that the effects of ES L1 antigens are restricted to differentiated DCs population, but not on their monocyte precursors. Although the physiological significance of this finding is still unclear and require independent investigation, such property of ES L1 could have evolved as a result of tight regulation of the host immune response by the parasite. This kind of modulation is aimed specifically to prevent the host immune response against the parasite itself, but it also mitigates the unwanted immune responses like those to autoantigens and allergens, consequently increasing the chances of host survival (37). Upon contact with ES L1 antigens, human monocyte-derived DCs acquired semi-mature phenotype, characterized by: low expression of HLA-DR, co-stimulatory molecules and CCR7; moderate expression of CD40; a clear anti-inflammatory cytokine profile, i.e., low production of IL-12 along with the enhanced production anti-inflammatory/regulatory cytokines, IL-10 and TGF-β, even after the challenge with pro-inflammatory stimuli. In line with this, an increased ratio of IL-10/IL-12 in DCs was found to contribute significantly to their tolerogenic functions and increased capacity to expand Tregs and downregulate differentiation of Th1 and Th17 cells (50, 61). CCR7 expression by IL-10-producing DCs was shown critical for their migration to lymphoid organs and polarization of naïve T cells into Tregs (62–64). Somewhat increased expression of CCR7 by ES L1-treated DCs and their tolerogenic potential are in line with these findings. High production of IL-10 and TGF-β could be responsible for the decreased expression of co-stimulatory molecules and blocking of IL-12 production by DCs, as suggested by other findings (59). Also, synergistic effects of IL-10 and TGF-β were shown to result in enhanced tolerogenic properties of DCs and their higher potential to induce IL-10-producing Tregs (65), which is in line with the properties of ES L1-treated DCs observed in this study.

The stability of tolerogenic DCs and preservation of anti-inflammatory phenotype in inflammatory surrounding is the most important issue when creating cell-based therapies for autoimmune diseases (61). TLRs were shown to be critically involved in the recognition of damage-associated molecular patterns (DAMPs), driving the unwanted inflammatory response in autoimmune diseases (66). In addition to TLR3, which was shown to be involved in the recognition of self-RNAs released from necrotic synovial fluid cells in rheumatoid arthritis patients (67), TLR4 has been implicated in the recognition of various DAMPs in different autoimmune processes (68). In line with this, ES L1-treated DCs displayed the resistance to the maturation induced by both TLR3 (Poly I:C) and TLR4 (LPS) agonist in particular. In contrast to Poly (I:C) and LPS, known to induce predominantly Th1 (56) and a mixed Th1/Th2 response (69), respectively, LPS/IFN-γ cocktail is known to induce inflammatory type 1 DCs (52), which are able to induce both Th1- and Th17-mediated immune response (50). Both, Th1 and Th17 cells were shown to be critically involved in the pathogenesis of chronic inflammation and autoimmunity (70). DCs maturated in the presence of ES L1 retained their tolerogenic properties regardless of activation with LPS/IFN-γ since, no noticeable change in the expression of surface markers and cytokine profile was observed. ES L1 antigens affected the maturation and cytokines production of DCs in a way that resembles tolerogenic DCs obtained under the influence of some immunosuppressive drugs (27, 28, 60) or immunosuppressive cytokines (65), but presumably *via* different mechanisms.

ES L1-treated DCs, even after the challenge with LPS/IFN-γ, exhibited a reduced capacity to induce T cell proliferation in mixed leukocyte reactions, which is in accordance with their observed semi-mature phenotype and tolerogenic functions. The experiments were performed using cells from healthy individuals. Due to HLA-restriction, the allogeneic coculture system applied in our study includes the response of a limited naïve T cell population that are able to respond to allogeneic MHC complexed with self-peptides (71). This model system may better reflect the potential response to ES L1-treated DCs *in vivo*, compared to models with a polyclonal stimulation of T cells, especially since recent findings suggested that *T. spiralis*-secreted components are structurally related to some human cell components (39), so only a limited repertoire of naïve T cells may be able to respond. Moreover, these DCs impaired inflammatory Th1 and Th17 cell responses manifested as both reduced production of IFN-γ and IL-17 cytokines and the percentage of Th1 and Th17 cells, respectively. Lower Th1 polarizing capacity of ES L1-treated LPS/IFN-γ-matured DCs might be a consequence of their low capacity to produce IL-12, a known Th1-polarizing factor (72). The fact that ES L1-treated DCs enhanced TGF-β production by T cells may also explain diminished production of Th1 polarization capacity and reduced allogenic proliferation, since TGF-β was shown to be critically involved in both processes (73). Although it is known that TGF-β contributes to regulatory as well as to Th17 type of immune response, there is evidence that low levels of TGF-β promote Th17 response, while increased TGF-β influences the elevated Foxp3 expression, hence promoting the expansion of Tregs (74). Since IFN-γ and IL-17 contribute in the genesis of autoimmune diseases (75, 76), the capacity of ES L1-induced tolerogenic DCs to reduce the production of these cytokines could favor the potential applicability of these DCs in the treatment of autoimmune diseases. The observations, that secretory products from different parasitic worms heavily skew the immune response toward Th2 type *via* DCs while inhibiting Th1 and Th17 responses (34), are in line with the finding that ES L1-primed DCs induced Th2 type response, as indicated by significantly expanded Th2 cells and elevated IL-4 production. In addition to lowering Th1 response, an increased Th2 polarization could also be related to moderately increased expression of CD40 on DCs upon stimulation with ES L1, as it was shown that CD40 is critically involved in the induction of Th2 cells by DCs, especially during helminths infection (77).

Besides the suppression of inflammatory immune response and the enhancement of Th2 type of response, ES L1-treated DCs demonstrated the ability to induce the expansion of CD4^+^CD25^hi^Foxp3^hi^ T regulatory cells. This expansion was reduced upon addition of 1-MT, an IDO-1 inhibitor (48) during the coculture, suggesting that Tregs inducing capacity of ES L1-treated DCs is IDO-1 dependent. IDO-1-mediated actions on the induction of Tregs include both deprivation of tryptophan and kynurenine-dependent induction of Tregs *via* aryl hydrocarbon receptor (78). An increased expression of IDO-1 in DCs additionally treated with LPS/IFN-γ could be explained by the fact that IFN-γ is a strong inducer of IDO-1 (79). The results from these experiments also suggested that IDO-1 is more involved in the induction of Tregs by ES L1-treated DCs matured with LPS/IFN-γ than DCs treated with ES L1 only, as a stronger inhibition of Treg induction was obtained with the former. Therefore, DCs treated with ES L1 could have utilized additional mechanisms besides IDO-1, such as IL-10- or ILT-3-mediated induction of Tregs, both of which were shown to be involved in the induction of Tregs by tolerogenic DCs (61). Tregs are involved in the suppression of effector T-cell activity and maintenance of immunologic self-tolerance, as underlying processes in the modulation of autoimmune diseases (80). The finding that Treg-inducing capacity of ES L1-treated DCs is retained, even after the challenge with strong pro-inflammatory stimuli, is in agreement with previous findings on tolerogenic DCs primed by immunomodulatory molecules or immunosuppressive drugs (27, 81). Tregs induced by ES L1-treated DCs upregulated the production of IL-10 which may be the consequence of high IL-10/IL-12 ratio observed in ES L1-treated DCs that induced the expansion of Tregs, as previous findings on the mechanisms of Tregs induction suggest (61, 82). The same population of Tregs showed an elevated expression of TGF-β, probably due to the increased production of IL-10 and TGF-β by DCs (61). Although both TGF-β and IL-10 exhibit immunosuppressive functions, they were also shown to negatively regulate each other (83, 84). These findings could partially explain why Tregs induced by ES L1-treated DCs expressed predominantly IL-10, whereas Tregs induced by LPS/IFN-γ-matured ES L1-treated DC produced predominantly TGF-β. It is also possible that different mechanisms of tolerogenic induction were triggered after the maturation of ES L1-treated DCs, but these hypotheses require further investigations. A relevant criterion for the evaluation of optimal DCs properties for tolerance induction is their capacity to induce Treg cells that are able to inhibit allogeneic T cell proliferation, and this property was observed in some tolerogenic DCs like those primed by IL-10 (31, 57). We showed here that T cells primed with ES L1-treated DCs successfully suppressed the proliferation of allogeneic PBMCs, probably due to increased prevalence of Tregs cells in resulting T cell population, as well as other suppressive T cell population induced by ES L1-treated DCs.

Using HEK-Blue™ reporter cells lines expressing individual TLR or NOD-like receptors, we identified for the first time TLR2 and TLR4 as receptors that interact and induce intacellular signaling after ligation of ES L1 antigens. These results are consistent with the studies that have shown the interaction of TLR2 or TLR4 with other helminth antigens. For example, lipid fractions and lysophosphatidylserine of helminth *Schistosoma mansoni* and lipid of *Ascaris lumbricoides* reacted with TLR2 on DCs and mediate their differentiation into cells that induce Th2 and regulatory immune responses (85, 86). Lacto-*N*-fucopentaose III of *S. mansoni* and ES 62 glycoprotein secreted by *Acanthocheilonema vitae*, were shown to activate TLR4 receptor and lead to consequent polarization of T cell responses toward Th2 (87, 88). Even though the microbial and helminthic products can engage the same TLRs, they can, most probably in combination with other PRRs, initiate different downstream signaling pathways that could lead to different immune response polarization. The precise underlying mechanisms are yet to be investigated.

The critical relevance of both TLR2 and TLR4 interaction with ES L1 antigens for the induction of tolerogenic properties in human DCs was demonstrated by blocking these two receptors before adding ES L1 antigens. Tolerogenic properties of ES L1-stimulated DCs were compromised when TLR2 and TLR4 were blocked, indicating, for the first time, that *T. spiralis* ES L1 antigens mediate phenotypical and functional maturation of DCs mainly *via* TLR2 and TLR4. The importance of both TLR2 and TLR4 was also demonstrated for the interaction with *S. mansoni* antigens (85, 89). Signaling *via* most TLRs normally results in the production of pro-inflammatory cytokines by DCs (90). However, helminth (and some microbial) products trigger Th2 and regulatory responses *via* interaction with TLRs (91). It is frequently suggested that stimulation of TLR2 is associated with Th2 immune response, while activation of DCs delivered through TLR4 results in Th1 type of response. ES L1 antigens interacted with both receptors on DCs, which resulted in the induction of anti-inflammatory responses. Moreover, ES L1 rendered DCs poorly responsive to TLR4-mediated induction of maturation, not *via* suppression of TLR4 expression, since we showed that ES L1 did not alter the expression of TLR4, but rather *via* modulation of downstream signaling events, and/or engagement of some other receptors expressed on DCs. Since ES L1 antigens are complex mixture of molecules, it is reasonable to assume that other PRRs are also involved in ES L1 recognition and binding. Indeed, it was revealed that ES L1 components are ligands for C-type lectin receptors, mannose receptor (92), and DC-SIGN (manuscript in preparation). Binding to lectin receptors could modulate intracellular signaling triggered by TLRs and affect DCs response in that way. Although we did not address this issue here, the results obtained with simultaneous blocking of TLR2 and TLR4 indicated that other receptors are involved as well. This presumption could also be supported by the finding that ES L1 did not affect DC differentiation, while exerted an impact on DCs maturation. The possible explanation for this phenomenon could be that completely differentiated DCs express different surface molecules compared to monocytes, including DC-SIGN, which could be important for the ES L1 impact on DCs. These findings open the possibilities for future research in trying to understand the mechanisms by which ES L1 induce toloregenic DCs.

In conclusion, this study revealed that the treatment of human monocyte-derived DCs with *T. spiralis* ES L1 antigens could be a promising new strategy for the development of stable tolerogenic DCs, with an increased capacity to suppress the inflammatory immune response while favoring the expansion of highly potent IL-10- and TGF-β- producing Tregs. Bearing in mind that the major scientific efforts are made to develop cell-based therapies that promote tolerance in humans, and although more investigation on mechanisms underlying the induction of tolerogenic DCs by ES L1 are needed, those tolerogenic DCs may present a potentially new tool for the treatment of inflammatory disorders.

## Ethics Statement

Animal experiments were performed according to institutional guidelines and were approved by the local Institutional Animal Care and Use Committee of the Institute for the Application of Nuclear Energy. Samples of human peripheral blood were obtained from healthy volunteers after written informed consent in accordance with the Declaration of Helsinki and approval by the Ethical Board of the Institute for the Application of Nuclear Energy.

## Author Contributions

LS-M, MC, NI, AG-M, ST, and EP participated in the design of the study. NI, AG-M, ST, DV, JC, and CA participated in data acquisition and analysis. NI, AG-M, and ST prepared the manuscript. LS-M, MC, ST, DV, and EP participated in data interpretation and manuscript revision. LS-M and EP supervised this study and they have equally contributed. All authors gave final approval of the version to be published; and agreement to be accountable for all aspects of the work in ensuring that questions related to the accuracy or integrity of any part of the work are appropriately investigated and resolved.

## Conflict of Interest Statement

The authors declare that the research was conducted in the absence of any commercial or financial relationships that could be construed as a potential conflict of interest. The handling Editor declared a past co-authorship with one of the authors LS-M.
