# Supplementary material for: Trichinella spiralis Excretory–Secretory Products Induce Tolerogenic Properties in Human Dendritic Cells via Toll-Like Receptors 2 and 4
Source: Front Immunol. 2018 Jan 24;9:11. doi: 10.3389/fimmu.2018.00011 (PMC5787699; doi:10.3389/fimmu.2018.00011)
Supplement: Supplementary file 1 [file Image_1.PDF]

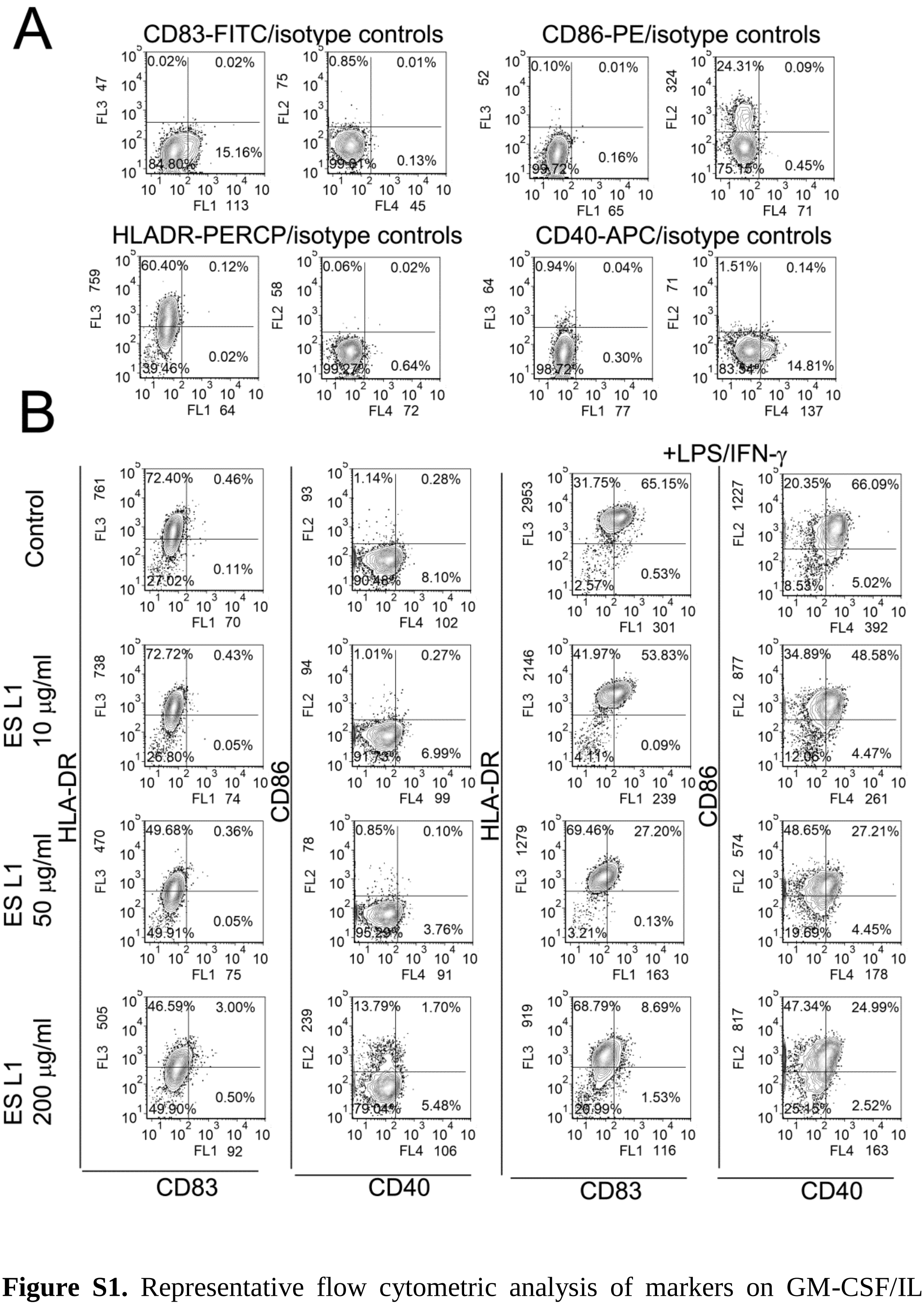

Figure S1. Representative flow cytometric analysis of markers on GM-CSF/IL-4-induced DCs, differentiated in the presence or absence of different doses of ES L1 antigen (10, 50 or 200 μg/ml) for 5 days, and then stimulated with LPS/IFN-γ, or left unstimulated, for the next 24h. **(A)** In each experiment, the collected cells were pooled and stained with one specific directly labeled antibody, and three isotype control antibodies labeled in different fluorochromes (FITC, PE, PERCP or APC), to set up the compensation parameters, and to determine the level of non-specific fluorescence for each FL channel. **(B)** Representative analysis from one experiment, out of three with similar results is shown (see also Figure 1C for summarized results).
