# Supplementary material for: Trichinella spiralis Excretory–Secretory Products Induce Tolerogenic Properties in Human Dendritic Cells via Toll-Like Receptors 2 and 4
Source: Front Immunol. 2018 Jan 24;9:11. doi: 10.3389/fimmu.2018.00011 (PMC5787699; doi:10.3389/fimmu.2018.00011)
Supplement: Supplementary file 2 [file Image_2.PDF]

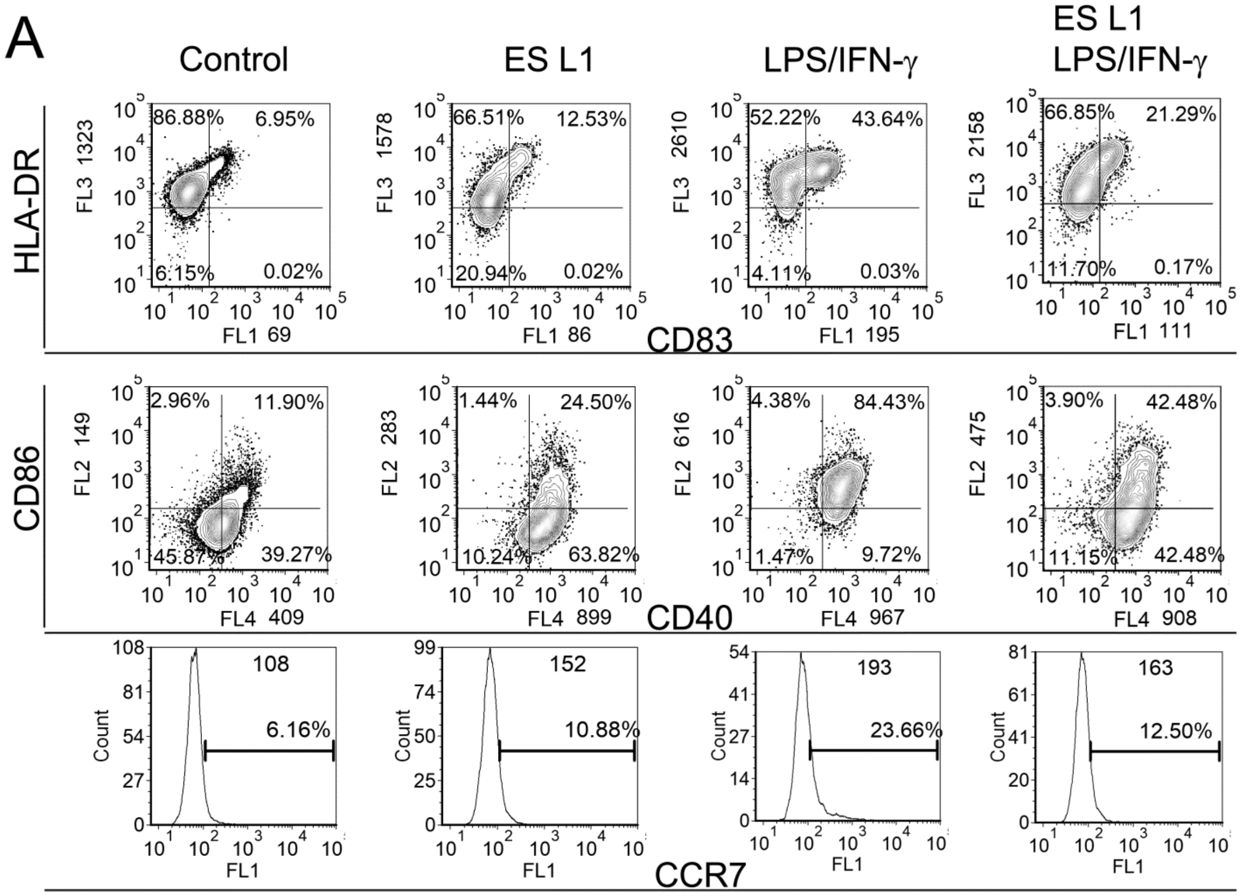

Figure S2. Representative flow cytometric analyses of surface markers expression on DCs treated or not with ES L1 (50 μg/ml) on day 4 of culture for 24h, and then stimulated with LPS/IFN-γ, or left unstimulated, for the next 24h. Representative plots for the expression of CD83, CD86, CD40, HLA-DR and CCR7 on DCs are shown from one out of four different experiments (See Figure 2A for summarized results). The specific fluorescence was determined according to single stained samples and isotype control antibodies, as described in Supplementary Figure 1.
