# Supplementary material for: Trichinella spiralis Excretory–Secretory Products Induce Tolerogenic Properties in Human Dendritic Cells via Toll-Like Receptors 2 and 4
Source: Front Immunol. 2018 Jan 24;9:11. doi: 10.3389/fimmu.2018.00011 (PMC5787699; doi:10.3389/fimmu.2018.00011)
Supplement: Supplementary file 4 [file Image_4.PDF]

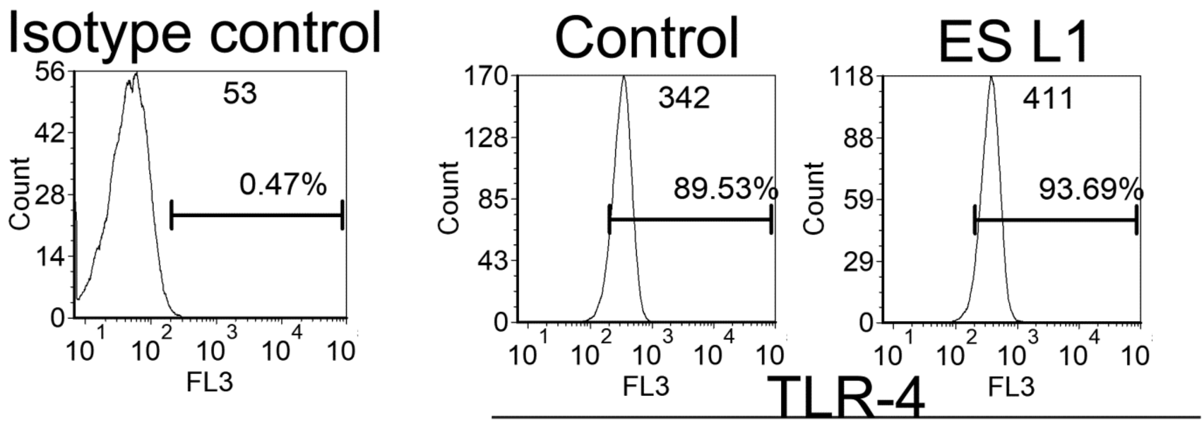

Figure S4. Expression of TLR-4 on ES L1 treated DC. The expression of TLR-4 was determined by flow cytometry on DCs that were treated or not with ES L1 (50 μg/ml) on day 4 of culture for the next 48 hours. Representative analysis is shown.
