# Supplementary material for: Trichinella spiralis Excretory–Secretory Products Induce Tolerogenic Properties in Human Dendritic Cells via Toll-Like Receptors 2 and 4
Source: Front Immunol. 2018 Jan 24;9:11. doi: 10.3389/fimmu.2018.00011 (PMC5787699; doi:10.3389/fimmu.2018.00011)
Supplement: Supplementary file 5 [file Image_5.PDF]

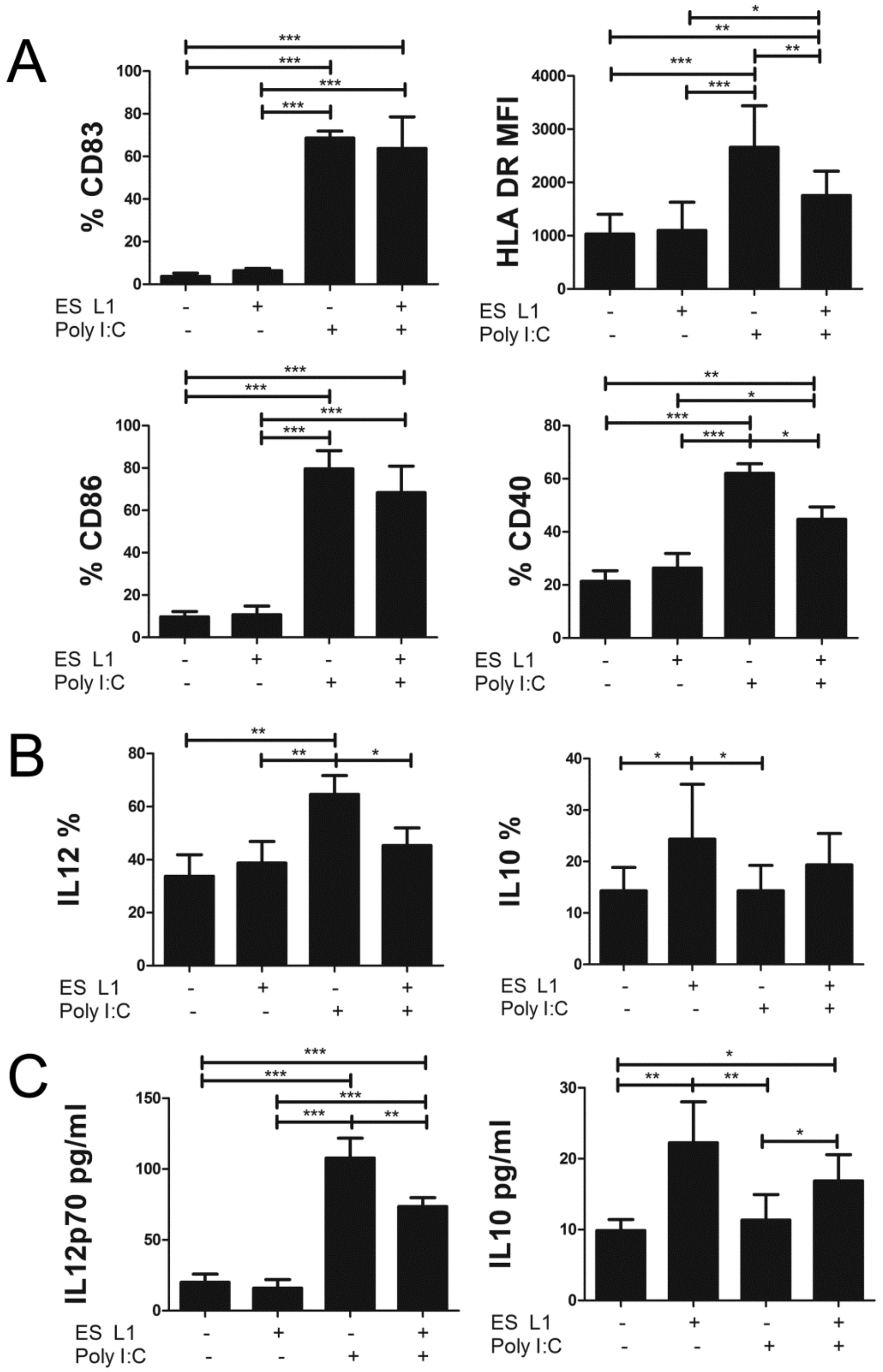

Figure S5. The maturation capacity of DCs treated with ES L1 antigens upon stimulation with Poly (I:C). **(A-C)** Immature DCs were treated with ES L1 antigens (50 μg/ml) on day 4 of culture for 24h, and then additionally activated or not with Poly (I:C) (10 μg/ml) for the next 24h, followed by flow cytometry analysis. **(A)** The surface expression of CD83, CD86, HLA-DR and CD40, and **(B)** the intracellular expression IL-10, IL12p40/p70 and TGF-β by DCs, were determined by flow cytometry and the results are shown as mean ± SD from three different experiments. **(C)** The levels of IL-10, IL-12p70 and TGF-β (pg/ml) in DCs culture supernatants were measured by ELISA test. * $p < 0.05$, ** $p<0.01$, *** $p<0.005$ as indicated (One-way ANOVA with Tukey post-test).
